# Supplementary material for: Complex‐centric proteome profiling by SEC‐SWATH‐MS
Source: Mol Syst Biol. 2019 Jan 14;15(1):e8438. doi: 10.15252/msb.20188438 (PMC6346213; doi:10.15252/msb.20188438)
Supplement: Supplementary file 8 — Dataset EV7 [file MSB-15-e8438-s008.zip › feature_plots_string/Q8NHY2.pdf]

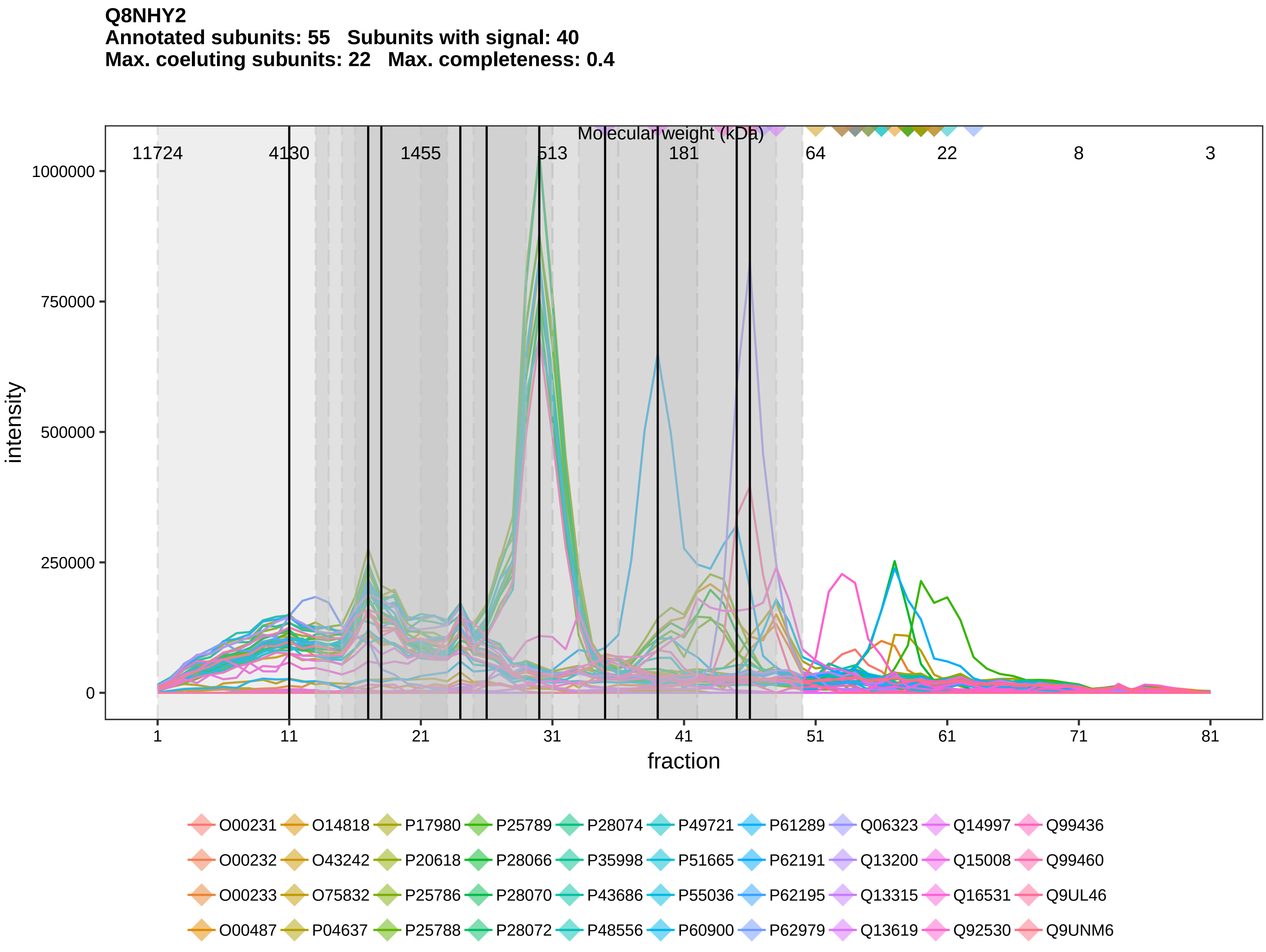
Q8NHY2
Annotated subunits: 55   Subunits with signal: 40
Max. coeluting subunits: 22   Max. completeness: 0.4
Molecular weight (kDa)
11724
4130
1455
513
181
64
22
8
3
intensity
1000000
750000
500000
250000
0
fraction
1
11
21
31
41
51
61
71
81
O00231
O14818
P17980
P25789
P28074
P49721
P61289
Q06323
Q14997
Q99436
O00232
O43242
P20618
P28066
P35998
P51665
P62191
Q13200
Q15008
Q99460
O00233
O75832
P25786
P28070
P43686
P55036
P62195
Q13315
Q16531
Q9UL46
O00487
P04637
P25788
P28072
P48556
P60900
P62979
Q13619
Q92530
Q9UNM6
